# Climate change induced habitat expansion of nutria (*Myocastor coypus*) in South Korea

**DOI:** 10.1038/s41598-022-07347-5

**Published:** 2022-02-28

**Authors:** Pradeep Adhikari, Baek-Jun Kim, Sun-Hee Hong, Do-Hun Lee

**Affiliations:** 1 Institute of Ecological Phytochemistry, Hankyong National University, 327, Jungang-ro, Anseong-si, Gyeonggi-do 17579 South Korea; 2 National Institute of Ecology, 1210 Geumgang-ro, Maseo-myeon, Seocheon-gun, Chungcheongnam-do 33657 South Korea; 3 School of Plant Science and Landscape Architecture, Hankyong National University, 327, Jungang-ro, Anseong-si, Gyeonggi-do 17579 South Korea

**Keywords:** Ecology, Zoology, Climate sciences, Ecology

## Abstract

The nutria, (*Myocastor coypus*), is a semiaquatic rodent native to the subtropical and temperate regions of South America. The species was introduced to South Korea for meat and fur production purposes and a wild population has become established. The species subsequently invaded aquatic ecosystems and destroyed aquatic vegetation and cultivated crops. Thus, it is essential to understand their current distribution and future range expansion for effective control and eradication strategies to reduce the risk of colonization into new regions. In this study, we used niche modeling procedure to identify potentially suitable habitats for *M. coypus* under current and future predicted climate change using the maximum entropy algorithm. We found that the main habitat area of *M. coypus* is expected to expand under a warming climate from ~ 4069 km^2^ in the southern and southeastern regions of South Korea, to the northern border of the country, with estimated ranges of 21,744 km^2^, 55,859 km^2^, and 64,937 km^2^ by 2030, 2050, and 2070, respectively. The findings of the present study assist in identifying the future distribution and potential dispersion routes of *M. coypus* in South Korea, which is important for informing the government regarding essential management actions plans at regional and local scales.

## Introduction

*Myocastor coypus* is a large, semiaquatic rodent indigenous to the subtropical and temperate regions of South America^1^ and is currently included on the International Union for Conservation of Nature (IUCN) list of the 100 worst invasive global species^2^. *M. coypus* was introduced to North America, Europe, Asia, and Africa for meat and fur production^3–5^; however, because of the decreasing demand and the consequential reduced market price, individuals were either released or escaped from farmhouses, and wild populations have since established in natural environments^5,6^. Subsequently, *M. coypus* have invaded aquatic ecosystems and caused large economic losses by destroying cultivated crops, aquatic vegetation, and tree species because of their aggressive foraging habits, in addition to burrowing-related damage to river banks, dykes, and irrigation canals^5–7^. Additionally, *M. coypus* causes various zoonotic diseases such as leptospirosis, trichinosis, and toxoplasmosis^6,8,9^.

In South Korea, *M. coypus* were first introduced from France in 1985 for breeding purposes; however, immature breeding techniques and poor maintenance resulted in mortality of all the imported individuals. *M. coypus* were again imported from Bulgaria in 1987, and breeding was successful with production peaking in 2001^3,7,10^. Since approximately 1999, *M. coypus* have been escaping from their breeding environments and have settled as wild populations in the Nakdong River basin and across water bodies in the southeastern regions of Chungcheongbuk and Jeju Provinces^3,7,10^. By 2012, their presence was recorded in 13 administrative districts (Ads) in South Korea^7^. *M. coypus* pose several threats to aquatic ecosystems and agriculture in South Korea, and destruction of aquatic plant habitat, consumption of endangered aquatic plants, and damaging cultivated crops adjacent to their habitats have been well documented; however, there has been no record of serious threats to irrigation canals or other physical structures in South Korea^3,7,10^.

Controlling *M. coypus* populations and inhibiting their range expansion are difficult because of their high fecundity, with ~ 2–3 litters per year and litter sizes of 1–12 individuals^11^. For instance; lethal control via systematic trapping and hunting has been successful at the local level and has been adopted in several countries in the European Union (EU), United States (US), and South Korea^6,7,12^. The South Korean government planned to capture *M. coypus* in response to public requests to protect agricultural crops and aquatic ecosystems, and a pilot project was conducted in 2013 which successfully captured 3349 individuals^7^. Subsequently, the Ministry of Environment established a 5-year Nutria Eradication Project in 2014, which was operated by a common cooperative system involving 18 organizations including the National Institute of Ecology, local governments, and local environmental agencies. The project facilitates public participation by providing incentives to the public when captured *M. coypus* are shown to local governments. The project had total operating funds of $9.5 million USD^7^. In total, 27,487 M*. coypus* individuals were captured between 2014 and 2018^7^. Accordingly, the distribution and habitat tracks (i.e., species occurrence within a 0.01 km^2^ area) of *M. coypus* within South Korea were investigated from 2014–2018 to provide baseline data for informing eradication projects.

The threats posed by *M. coypus* can be exacerbated by changing climates and the correlated expansion of suitable habitat, which enhances reproduction and survival success^13,14^. Extreme cold winter temperatures have been suggested to limit *M. coypus* distribution to areas with ≤ 80 annual freezing days per year^15^; thus, increasing global temperatures owing to climate change could favor *M. coypus* habitat expansion^14^. In South Korea, the mean temperature has increased by 1.8 °C over the last 100 years, and is predicted to increase by an additional 0.63 °C every decade until 2100, with the increase reaching > 4.7 °C by 2100^16^ and potentially creating a highly favorable environment for the spread of invasive species in South Korea.

Successful management of *M. coypus* requires an adequate understanding of their ecology and distribution^13^. In particular, assessing *M. coypus* distribution under current and projected climate and land-cover change scenarios can help prioritize management and monitoring regions for early detection, in addition to informing the preventative measures adopted by governments and conservationists. Therefore, it is essential to develop reliable methods for predicting future *M. coypus* invasion so that inspection or management policies can be adjusted to decrease the risk of establishment, propagation, and expansion into new areas.

Species distribution models (SDMs) are an empirical tool used in ecological studies to estimate the potential spatial distributions of species in response to climate change, and determine the ecological niches of species based on records of species occurrences and environmental variables^17^. Among the various available SDM algorithms, maximum entropy (MaxEnt) is a machine-learning technique with high predictive performance based on a small subset of species presence data^18^. Although various studies have examined *M. coypus* in South Korea, to date most have focused on the introduction, ecology, distribution, diet, and adverse impacts of *M. coypus* on aquatic ecosystems and agriculture^3,7,10,19–21^. To the best of our knowledge, a detailed investigation of their distribution considering bioclimatic factors, land cover, altitude, and distance from water has not previously been conducted. Accordingly, the main objectives of the present study were to: (1) Conduct an extensive survey of *M. coypus* across South Korea; (2) analyze the associations between bioclimatic, land cover, geographic, and water proximity variables and current *M. coypus* distributions; and (3) model the potential *M. coypus* habitat under current and future climate and land-cover change scenarios in South Korea. Based on this study, high risk locations for *M. coypus* invasion were identified. The results can assist with understanding the distribution and potential future dispersion of *M. coypus* in South Korea, and support the development of a theoretical framework for management policies and strategies to control their potential spread.

## Results

### Species occurrence survey

**Figure 1:**
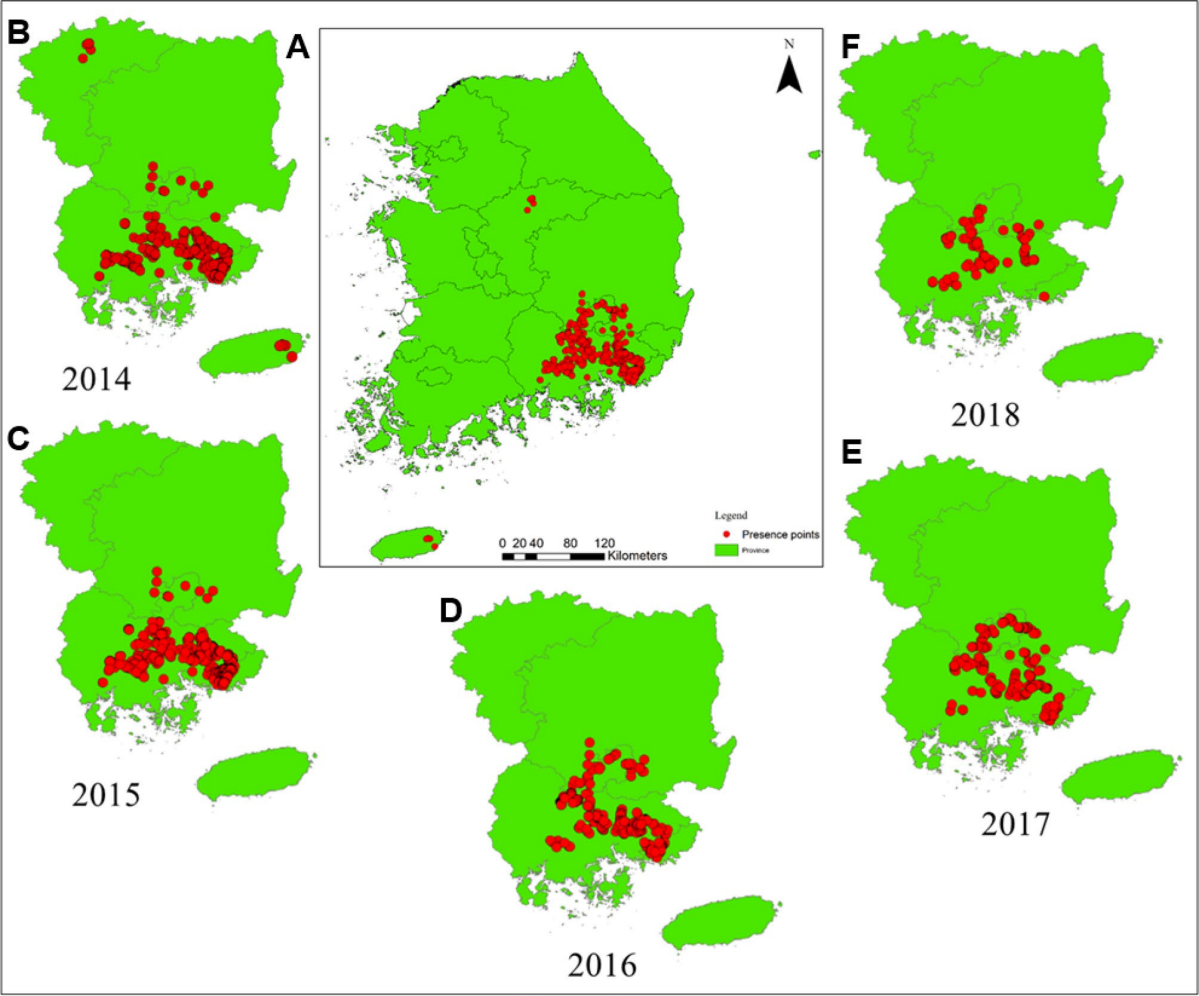
Nutria (*Myocastor coypus*) occurrence records in South Korea from 2014‒2018. (**A**) A total of 6,180 occurrence records were made in six administrative divisions including Chungcheongbuk, Gyeongsangbuk, Daegue, Gyeongsangnam, Busan, and Jeju. *M. coypus* survey records in: (**B**) 2014 (3,805 points), (**C**) 2015 (1,451 points), (**D**) 2016 (502 points), (**E**) 2017 (235 points), and (**F**) 2018 (187 points). Species occurrence was recorded in six ADs (Chungcheongbuk, Gyeongsangbuk, Daegue, Gyeongsangnam, Busan, and Jeju) in 2014, and in four ADs (Gyeongsangbuk, Daegue, Gyeongsangnam, and Busan) from 2015‒2018. Generated using ArcGIS Desktop 10.8 (https://desktop.arcgis.com).

A species occurrence survey was conducted to support the “*M. coypus* Eradication Project” established by the Ministry of Environment, South Korea, from March 2014 to December 2018. Based on the field survey and habitat track study, the highest number of *M. coypus* was recorded in 2014 (3,805 counts) and successively declined thereafter; i.e., to 1,451 in 2015, 502 in 2016, 235 in 2017, and 187 in 2018 (Fig. 1). In 2014, *M. coypus* were detected in 19 local administrative districts (LADs) across six ADs: Chungcheongbuk (2 LADs), Jeju (2 LADs), and local ADs of Gyeongsangbuk, Daegue, Gyeongsangnam, and Busan (15 LADs). The main habitat was in the midstream and downstream regions of the Nakdong River basin, between Busan and Daegu. The numbers of habitat track were detected in 15 LADs between 2015 and 2017, and in 14 LADs in 2018. These results showed an estimated 54% reduction in *M. coypus* habitat tracks between 2016 and 2017, demonstrating progress in eradication following implementation of the *M. coypus* eradication project.

### Model performance and variable importance

Pearson’s correlation test was performed, and based on the weak correlations (r < 0.70), five bioclimatic variables (e.g., annual mean temperature [Bio 1], maximum temperature of the warmest month [Bio 5], mean temperature of the wettest quarter [Bio 8], precipitation seasonality [Bio 15], and precipitation of the wettest quarter [Bio 16]) and three environmental variables (i.e., altitude, distance from water, and land-cover change; Table S1) were selected and used for MaxEnt modeling. A jackknife test was performed to confirm the contribution of the included variables in the model (Fig. S1) and the results showed that the temperature-related variables Bio 5, Bio 8, and Bio 1 were the strongest bioclimatic predictors of the *M. coypus* distribution, explaining 29.23%, 21.05%, and 15.01% of the variance, respectively (Table 1). Similarly, both altitude and distance from water played relatively important roles, explaining 14.3% and 8.25% of the observed variance, respectively. Other variables had markedly lower values, thus appearing to play more minor roles in the *M. coypus* distribution model. The area under the curve (AUC) values of the (training 0.968 and test 0.954) indicated a high model performance for the prediction of the *M. coypus* distribution. Similarly, the true skill statistic (TSS) values (0.910) showed strong agreement between the observations and modeled predictions.

**Table 1:** Contributions of environmental variables to the species distribution model of *M. coypus* in South Korea calcualted using a jackknife test.

Code	Environmental variable	Contribution (%)
Bio05	Max. temperature of warmest month	29.23
Bio08	Mean temperature of wettest quarter	21.05
Bio01	Annual mean temperature	15.01
dem	Altitude	14.3
dwater	Distance from water	8.25
Bio16	Precipitation of wettest quarter	4.51
Bio15	Precipitation seasonality	4.32
ssp1	Land-cover change	3.12

### Predictions of current and future distributions of *M. coypus*

**Figure 2:**
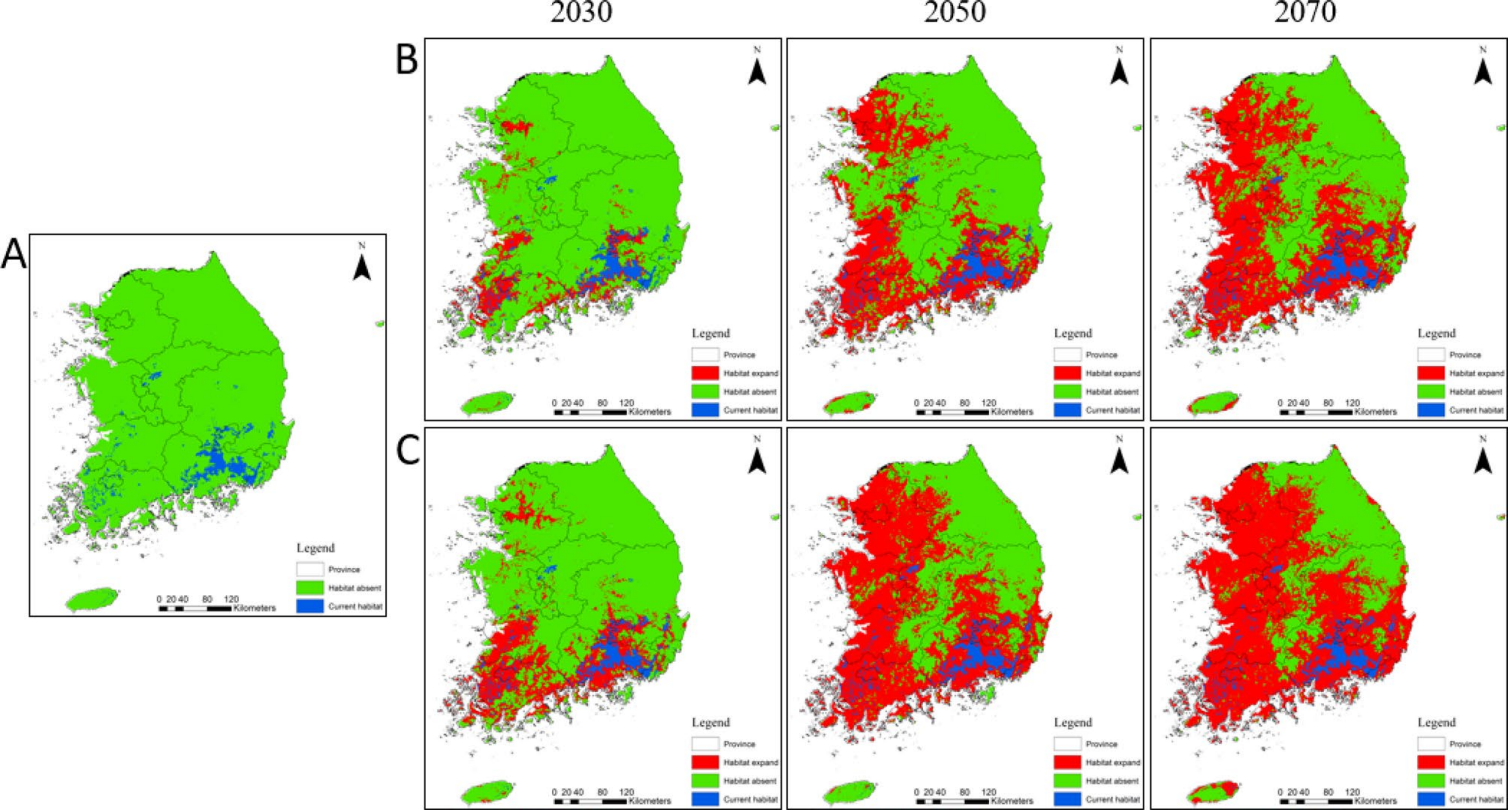
Prediction of binary distribution map of *Myocastor coypus* in South Korea. Blue, red, and green indicate the prediction of current habitat, habitat expansion in the future (2030, 2050, and 2070), and the absence of *M. coypus* habitat, respectively. (**A**) Current potential habitat, and future potential habitat under the climate change scenarios (**B**) RCP 4.5 and (**C**) RCP 8.5. Generated using ArcGIS Desktop 10.8 (https://desktop.arcgis.com).

**Figure 3:**
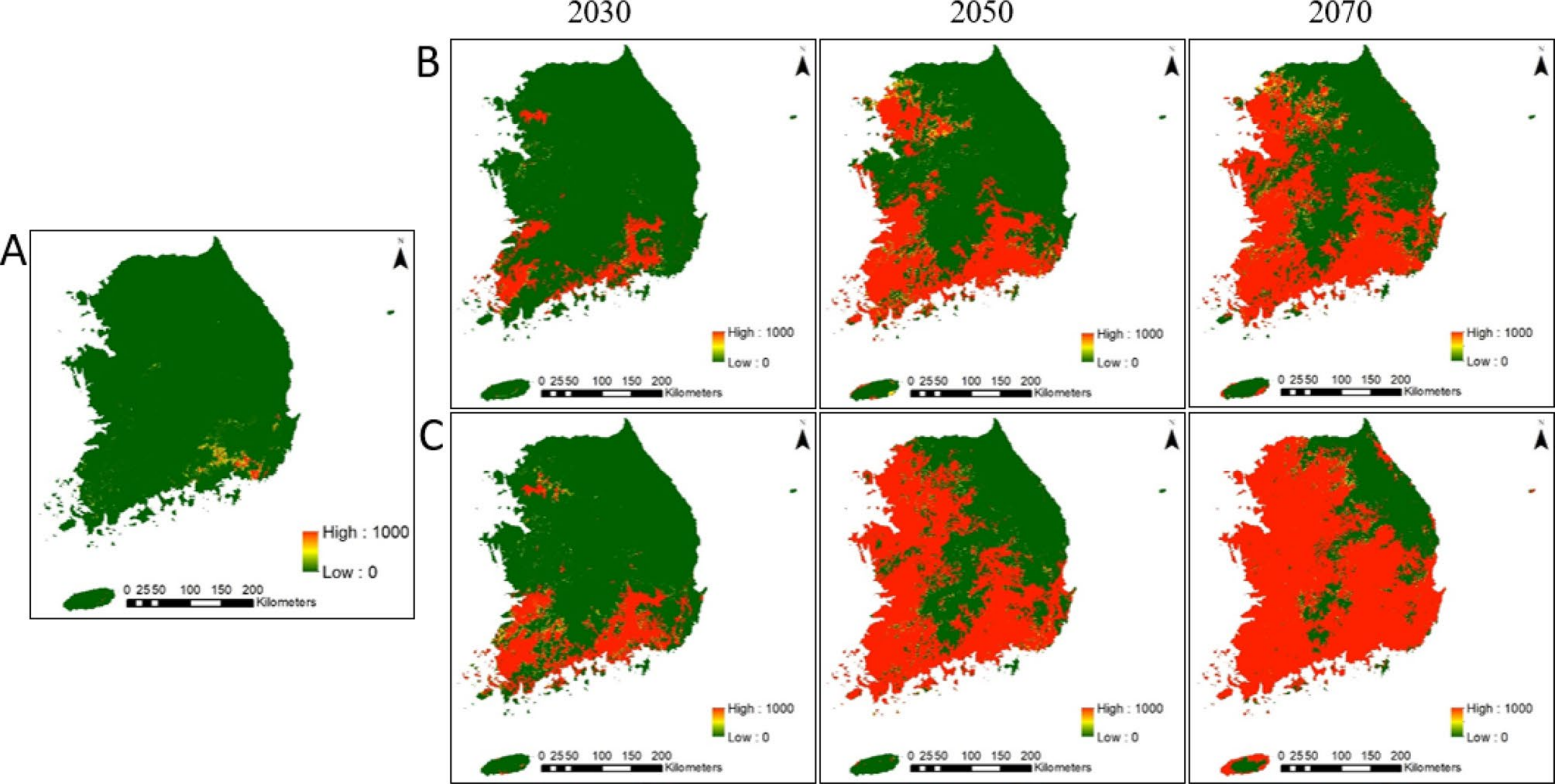
Prediction of habitat suitability map of *Myocastor coypus* in South Korea. The suitability maps ranged from 0‒1000 under (**A**) the current climate, and future climate change scenarios (**B**) RCP 4.5 and (**C**) RCP 8.5 in 2030, 2050, and 2070. Generated using ArcGIS Desktop 10.8 (https://desktop.arcgis.com).

The extent of climatically suitable *M. coypus* habitats was modeled to show the distribution and the potential habitat area across all South Korean ADs under current and future climate change scenarios RCP 4.5, and RCP 8.5^22^ in 2030, 2050, and 2070 (Table 2). The current potential *M. coypus* habitat was identified in 12 provinces (Fig. 2A), with a total area of ~ 4,069 km^2^, equivalent to 4.26% of the total country land area. The seven provinces concentrated along the southern and southeastern regions of the country, i.e., Gyeongsangnam (2,211 km^2^), Jeollanam (506 km^2^), Gyeongsangbuk (498 km^2^), Busan (188 km^2^), Daegu (213 km^2^), Chungcheongbuk (160 km^2^), and Jeollabuk (142 km^2^), were predicted to be highly suitable for *M. coypus* habitation (Table 2). Suitability maps are independent of thresholds and commonly represent the probability of focal species to occur in a given geographical area. Therefore, we predicted the probability distribution maps of *M. coypus* and presented the current suitable habitats in Fig. 3A. Under the current climate, both binary distribution maps and suitability maps showed consistent predictions for *M. Coypus* habitation. We calculated the mean and standard deviation of suitability values in each AD, indicating that the maximum mean suitability value under the current climate was in Busan (0.23, standard deviation = 0.40) (Table 3).

**Table 2:** Potential distribution area (km^2^) of *Myocastor coypus* in different administrative divisions of South Korea predicted using a species distribution model under the climate change scenarios RCP 4.5 and RCP 8.5 for the years 2030, 2050, and 2070.

Administrative division		RCP 4.5			RCP 8.5		
	Current	2030	2050	2070	2030	2050	2070
Seoul	0	385	563	577	399	595	600
Incheon	0	12	458	501	1	560	614
Gangwon	0	0	540	1,600	51	2,176	3,298
Gyeonggi	0	495	5,670	7,277	1,271	9,023	9,454
Sejong	5	4	160	334	26	397	437
Chungcheongbuk	160	32	673	1,951	93	3,329	4,430
Daejeon	0	39	321	469	61	479	526
Chungcheongnam	29	448	2,410	6,147	437	6,685	7,223
Daegu	213	563	723	803	660	801	842
Jeollabuk	142	1,441	4,745	5,314	3,522	5,439	6,251
Gyeongsangbuk	498	839	3,127	7,425	2,154	7,963	10,913
Gwangju	48	383	475	480	461	481	484
Ulsan	46	33	477	403	175	613	884
Busan	188	32	604	520	286	610	635
Gyeongsangnam	2,211	3,116	6,501	7,428	5,722	7,344	8,122
Jeollanam	506	3,452	8,324	8,692	6,340	9,293	9,583
Jeju	23	61	215	278	85	71	641

**Table 3:** Mean suitability and standard deviation (SD) of *M. coypus* in different administrative divisions (ADs) of South Korea under the climate change scenarios RCP 4.5 and RCP 8.5.

AD	Current		RCP 4.5						RCP 8.5					
			2030		2050		2070		2030		2050		2070	
	Mean	SD	Mean	SD	Mean	SD	Mean	SD	Mean	SD	Mean	SD	Mean	SD
Seoul	0.00	0.00	0.58	0.49	0.90	0.29	0.93	0.25	0.56	0.48	0.95	0.20	0.98	0.14
Incheon	0.00	0.00	0.01	0.09	0.60	0.46	0.75	0.42	0.00	0.04	0.87	0.33	1.00	0.00
Gangwon	0.00	0.00	0.00	0.00	0.01	0.09	0.06	0.22	0.00	0.01	0.08	0.27	0.29	0.44
Gyeonggi	0.00	0.00	0.02	0.13	0.43	0.46	0.62	0.46	0.04	0.18	0.81	0.38	0.96	0.20
Sejong	0.00	0.00	0.00	0.00	0.24	0.42	0.65	0.47	0.00	0.01	0.84	0.36	1.00	0.05
Chungcheongbuk	0.00	0.02	0.00	0.01	0.04	0.18	0.19	0.38	0.00	0.03	0.39	0.48	0.79	0.40
Daejeon	0.02	0.07	0.59	0.48	0.80	0.39	0.89	0.30	0.74	0.44	0.90	0.29	0.98	0.15
Chungcheongnam	0.00	0.00	0.01	0.09	0.23	0.41	0.74	0.42	0.01	0.07	0.84	0.36	0.99	0.09
Daegu	0.00	0.00	0.05	0.23	0.55	0.48	0.84	0.35	0.09	0.29	0.85	0.35	1.00	0.00
Jeollabuk	0.00	0.01	0.16	0.37	0.56	0.48	0.64	0.47	0.37	0.46	0.66	0.46	0.88	0.32
Gyeongsangbuk	0.00	0.04	0.03	0.18	0.15	0.35	0.34	0.47	0.08	0.26	0.37	0.48	0.72	0.44
Gwangju	0.01	0.02	0.70	0.44	0.97	0.17	0.98	0.13	0.94	0.24	0.98	0.13	1.00	0.06
Ulsan	0.01	0.10	0.02	0.14	0.39	0.47	0.36	0.47	0.15	0.35	0.57	0.49	0.89	0.31
Busan	0.23	0.40	0.01	0.09	0.83	0.35	0.75	0.42	0.37	0.47	0.90	0.27	0.92	0.26
Gyeongsangnam	0.07	0.21	0.29	0.45	0.64	0.47	0.75	0.43	0.54	0.49	0.74	0.43	0.88	0.33
Jeollanam	0.01	0.06	0.31	0.46	0.78	0.40	0.85	0.35	0.55	0.48	0.91	0.28	0.96	0.19
Jeju	0.01	0.07	0.02	0.10	0.10	0.29	0.15	0.35	0.04	0.19	0.03	0.17	0.53	0.49

The mean suitability value in each AD ranged from 0‒1. SD, standard deviation.

**Figure 4:**
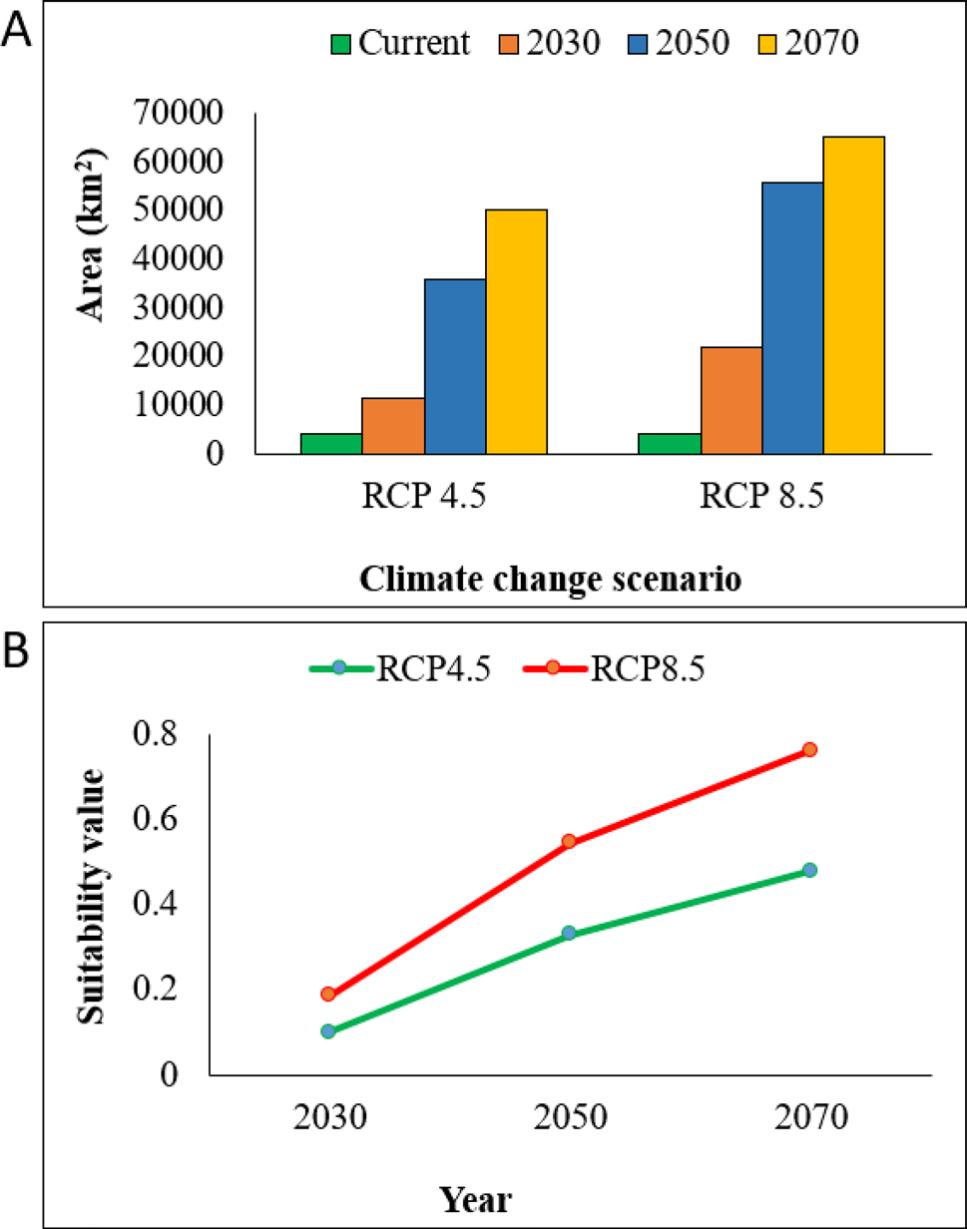
Estimates of the potential distribution area and mean suitability of *Myocastor coypus* under current and future predicted climate change in South Korea. (**A**) Suitable habitat areas by 2030, 2050, and 2070; (**B**) comparison of the mean suitability between the pair of years under the climate change scenarios RCP 4.5 and RCP 8.5.

Under climate change projections, the *M. coypus* habitat range was predicted to expand northward along the western and eastern coasts of the Korean Peninsula, spreading to all provinces by 2050 (Figs. 2B,C, 3B,C). The future suitable habitat areas were estimated to be 11,335 km^2^, 35,986 km^2^, and 50,199 km^2^ by 2030, 2050, and 2070, respectively, under RCP 4.5, and 21,744 km^2^, 55,859 km^2^, and 64,937 km^2^, respectively, under RCP 8.5 (Fig. 4A). Similarly, the mean suitability between the RCP 4.5 and RCP 8.5 for the years 2030, 2050, and 2070 was highest under RCP 8.5, suggesting that a warming climate will favor *M. coypus* habitation (Fig. 4B). Although the rates and trends of habitat expansion were inconsistent, suitable habitats were expected to increase across all provinces (except the cities of Ulsan and Busan) by 2030 under RCP 4.5. The areas with the highest suitability were predicted to be in Jeollanam, Gyeongsangnam, Gyeongsangbuk, Chungcheongnam, and Gyeonggi by 2030, 2050, and 2070 under both examined climate change scenarios (Table 2). However, the mean suitability value was estimated to be the highest in Gwangju City where it was predicted to range from *P* = 0.70–1.00 between 2030 and 2070 (Table 3). The ADs located on the west coast (i.e., Jeollabuk, Chungcheongnam, and Gyeonggi) maintained higher rates of habitat expansion compared to those in the central region and along the east coast. Gangwon Province (northeastern region) and Jeju (southern region) had comparatively small areas of suitable habitat. In addition, high mountain regions (e.g., the Baekdu‒daegan mountain range and Hall Mountain of Jeju [elevation, 1950 m]) also exhibited low habitat suitability (Fig. 3).

### Vulnerability estimations of different administrative divisions

We performed vulnerability estimation of different ADs based on the mean habitat suitability values of *M. coypus* in different provinces, which revealed that all the ADs were considered to have low vulnerability. However, in the future, vulnerability will be extended from the southern region to central and northern regions and high vulnerability with be exhibited in two ADs (e.g., Daejeon and Gwangju) by 2030, eight ADs (e.g., Seoul, Incheon, and Daejeon) by 2050, and 11 ADs (e.g., Seoul, Incheon, and Gyeonggi) by 2070 (Table 4).

**Table 4:** Vulnerability of different administrative divisions (ADS) based on the mean suitability values estimated under RCP 4.5 and RCP 8.5 climate change scenarios.

Vulnerability	Current	2030	2050	2070
Low	Seoul, Incheon, Gangwon, Gyeonggi, Sejong, Ulsan, Chungcheongbuk, Daejeon, Busan, Chungcheongnam, Daegu, Jeollabuk, Gyeongsangbuk, Gwangju, Jeju Gyeongsangnam, Jeollanam,	Incheon, Gangwon, Gyeonggi, Sejong, Chungcheongbuk, Chungcheongnam, Daegu, Jeollabuk, Gyeongsangbuk, Ulsan, Busan, Jeju	Gangwon, Chungcheongbuk, Gyeongsangbuk, Jeju	Gangwon, Jeju
Moderate		Seoul, Gyeongsangnam, Jeollanam	Gyeonggi, Sejong, Chungcheongnam, Jeollabuk, Ulsan,	Chungcheongbuk, Gyeongsangbuk, Ulsan
High		Daejeon, Gwangju	Seoul, Incheon, Daejeon, Daegu, Gwangju, Busan, Gyeongsangnam, Jeollanam	Seoul, Incheon, Gyeonggi, Sejong, Daejeon, Busan, Chungcheongnam, Daegu, Jeollabuk, Gwangju, Gyeongsangnam

Low, moderate, and high vulnerability were classified according to the mean suitability values in the ranges from 0‒0.33, 0.34‒0.66, and 0.67‒1.00, respectively.

## Discussion

A 5-year field survey was performed to investigate the *M. coypus* distribution in South Korea, including identifying their presence in regions of the Nakdong River basin, Han River, and Jeju Island. The majority of habitat tracks were observed in the midstream and downstream regions of the Nakdong River basin, thus identifying this basin as the area that was the most populated by *M. coypus*. The midstream and downstream reaches of the Nakdong River are characterized by relatively slow water speeds upstream of several tributaries and wetlands^10^, warm winter temperatures, and abundant food sources, creating appropriate habitats for *M. coypus* growth and establishment^3^. Although it was observed in this study that the *M. coypus* habitat distribution was reduced to 14 LADs along the Nakdong River in 2018, their presence has continued in this area despite several management efforts, including the *M. coypus* Eradication Project^7^. However, there were no recorded sightings in either Jeju or Chungcheongbuk Province after 2014, implying significant population declines following the implementation of the eradication project. Globally, temperature is the most importnat factor for biological invasions^14^. In particular, *M. coypus* are warm-adapted species that are vulnerable to colder weather; therefore, they tend to propagate rapidly in areas with warm winters^23^. *M. coypus* populations can decline owing to adverse effects in natural environments with temperatures ranging between 0–5 °C^14^. In extreme cold, they fail to reproduce and often show high mortality (≤ 71%) when sheet ice forms on water bodies^1,24,25^. The Nakdong River, located in the southeastern region of South Korea, maintains relatively warm temperatures year-round, and has notably warm winters compared to the rest of the country; whereas, the other major rivers, such as the Han and Geum Rivers, are located in cold-temperature and temperate regions, respectively, with recorded winter temperatures < − 26.2 °C (KMA 2021, unpublished data). Although Hilts et al.^14^ suggested that *M. coypus* can persist where there are ≤ 80 annual freezing days, their presence was notably undocumented along the Han and Geum Rivers. The extreme cold winter water temperatures could explain the absence of *M. coypus* in these locations; however, these areas could potentially become suitable habitats under increasing temperatures in future^3,26^.

As previously stated, the Nakdong River is currently the most suitable habitat for *M. coypus* in South Korea. Increasing temperatures facilitate invasive species growth, range expansion, and naturalization^27^, while simultaneously reducing the resilience of ecosystems to exotic species^14^. The model used here predicted that *M. coypus* will retain their current distribution and that additional habitat expansion will occur in the future, which is supported by the findings of Hilts et al*.*^14^ and Pereira et al*.*^28^. Climatic factors generally have a dominant influence on species distribution at broad spatial scales^29^. The findings in the present study show that temperature-related variables are important determinants of climate-based *M. coypus* habitats (similar to the findings of Schertler et al.^13^. The results also predict habitat expansion from the southeastern region (particularly Gyeongsangnam, Jeollanam, Gyeongsangbuk, Busan City, and Daegu City) toward the central and northern regions of the country, which, in addition to Jeollabuk, Chungcheongnam, Chungcheongbuk, Gyeonggi, and Seoul City, were predicted to become highly vulnerable in future decades. These results support previous findings that show that increasing temperatures will expand the invasion risk northwards^30^, in addition to earlier projections indicating habitat expansion of invasive species with increasing temperatures^13,14,31^. Future expansion of the suitable *M. coypus* habitat is likely to occur gradually from south to north through the water channel connections of the five major rivers in South Korea. Northward expansion is expected to occur mainly from the west coast. The terrain of South Korea is characteristically low along the west coast and high along the east coast^31^. Consequently, most major rivers flow westward into the Yellow Sea and are relatively long, maintaining smooth slopes and wide basins. In contrast, the rivers flowing into the East Sea are relatively short and steeply sloped. The geographic terrain and river systems along the west coast, combined with increasing future temperatures, could favor the expansion of *M. coypus* habitat, supporting the future establishment of *M. coypus* populations. The derived model used in this study did not predict *M. coypus* habitats in high mountains or on sharp elevational gradients, such as the Baekdu‒daegan mountain range (701 km), Mt. Halla on Jeju Island (1,950 m), the northern part of Gyeongsangbuk Province, and most of Gangwon Province (as similarly shown by Sheffels^23^ and Pereira et al*.*^28^). These areas could thus serve as a geographical barrier, blocking any future dispersion. In addition, high-elevational areas experience extremely cold weather in winter, maintaining snowpack for long time periods, thus creating inhospitable conditions for *M. coypus*^15^.

In addition to bioclimatic variables related to local-scale features, habitat covariates, such as a high proportion of freshwater and forested shrub wetlands (i.e., marshlands or swamps) close to other wetlands, appeared likely to support increasing *M. coypus* populations^14^. Freshwater swamps are relatively productive systems that enhance high floral diversity, including densely layered understories of herbaceous plants, shrubs, young trees, and overstory trees^32^, possibly generating foraging habitat and thermal protection during extremely cold winters. *M. coypus* tend to prefer various aquatic plants (e.g., *Paspalum disthichum*, *Panicum tricholaenoides*, *Lemna minor*, *Spirodela polyrrhiza*, *Carex* spp., and *Schoenoplectus* spp.), depending on their habitat and the season^33,34^. In South Korea, Hong et al.^19^ reported *M. coypus* diets consisting of similar aquatic plants in the families of Poaceae, Cyperaceae, and Salviniacease, with some terrestrial plants belonging to the families of Chenopodiaceae, Asteraceae, and Amaranthaceae. Freshwater swamps, particularly those connected to groundwater, freshwater rivers, and lakes, could be appropriate *M. coypus* habitats considering the preference of this species for foraging near the edges of slow-speed water bodies, potentially maintaining connectivity between rivers and wetlands, facilitating dispersion and range expansion^10,21,35^. *M. coypus* can travel ≤ 5–6 km overnight^36^, and Aliev^37^ reported *M. coypus* dispersals of ≤ 120 km in eastern Europe within 2 years. Similarly, Hong et al.^3^ reported that the dispersal distance of *M. coypus* increased by 42.6 km between 2007 and 2008 in South Korea. Human activities (e.g., landscape fragmentation) are additional important factors affecting *M. coypus* occurrence^7^. Similarly, human disturbances (e.g., urban population density and distance to human settlements) are thought to be additional important factors affecting *M. coypus* distribution^7,38^. These results highlight the importance of local environmental factors in addition to bioclimatic variables for *M. coypus* dispersal and range expansion.

*M. coypus* can cause large-scale damage to aquatic ecosystems, with significant effects on crops and riverine vegetation^39^, potentially resulting in shortages of food resources for other wild herbivores inhabiting areas adjacent to *M. coypus* habitat (e.g., roe deer)^40^. *M. coypus* can weaken dams and irrigation systems by burrowing on riverbanks, thereby creating direct economic losses, as well as indirect losses connected to floods. Accordingly, the Ministry of Environment in South Korea conducted the *M. coypus* Eradication Project from 2014–2018, which was considered to have successfully reduced the range of *M. coypus* habitats in Jeju and Chungcheongbuk Provinces; however, major habitats persisted in the Nakdong River.

Based on the results of the present study, future suitable *M. coypus* habitats are likely to expand from the southeastern region to the northern international border. Currently, all the ADs are under the low vulnerable; however, by 2070, 11 ADs including Gwangju, Jeollabuk, Busan, Daegu, and Seoul, will be considered to have high vulnerability. The continuous *M. coypus* habitat expansion has already had adverse impacts on aquatic ecosystems and agriculture within South Korea, and the SDM-based prediction of *M. coypus* distribution provides a valuable tool for targeting areas with the highest risk of invasion. The results can assist with understanding *M. coypus* distribution and dispersion in South Korea, in addition to informing government and conservation agencies regarding management action plans and policies aimed at controlling or eradicating *M. coypus* at regional and local scales.

## Methods

### Study area

**Figure 5:**
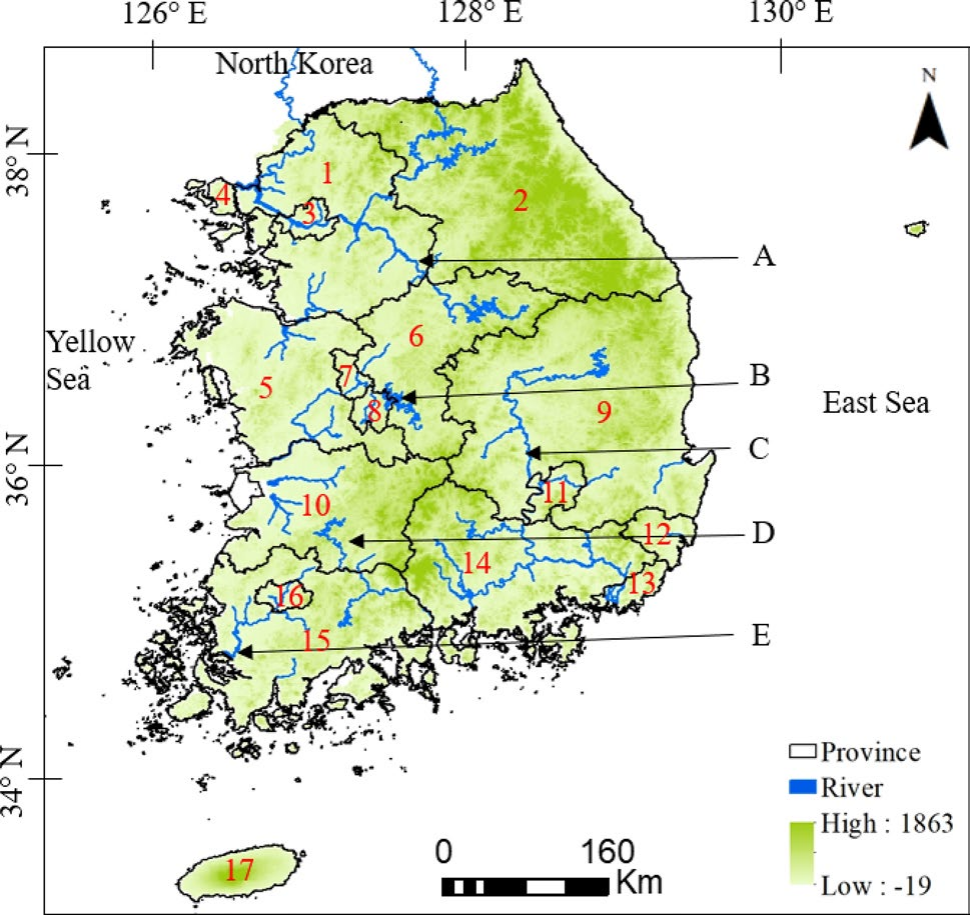
Elevation map of the study area, including the 17 provinces of South Korea: (1) Gyeonggi Province, (2) Gangwon Province, (3) Seoul City, (4) Incheon City, (5) Chungcheongnam Province, (6) Chungcheongbuk Province, (7) Sejong City, (8) Daejeon Province, (9) Gyeongsangbuk Province, (10) Jeollabuk Province, (11) Daegu Province, (12) Ulsan City, (13) Busan City, (14) Gyeongsangnam Province, (15) Jeollanam Province, (16) Gwangju City, and (17) Jeju City and six major rivers of South Korea: (**A**) Namhan River, (**B**) Geum River, (**C**) Nakdong River, (**D**) Seomjin River, (**E**) Nam River, and (**F**) Yeongsang River. Generated using ArcGIS Desktop 10.8 (https://desktop.arcgis.com).

The study area included the mainland and all the islands of South Korea, with a total land mass of 100,033 km^2^ (Fig. 5). The country is divided into 17 ADs (provinces and metro cities, referred to here as cities), which were utilized to assist in delineating *M. coypus* distribution localities and informing provincial governments when adopting future management actions. Geographically, the terrain of South Korea is mostly mountainous, dominating the northern and eastern parts of the country, whereas lowlands and flat plains occupy the southern and western regions. The Baekdu-daegan mountain range spans ~ 701 km, starting from Hyanro-bong in Goseong County of Gangwon Province and ending at Cheonwang-bong on Mt. Jiri in Jeollanam Province. The climate of South Korea is readily divisible into warm-temperate (southern coast and islands), temperate (northern and central), and cold-temperate (high mountains)^41^. Mean winter temperatures range between − 6 and 3 °C, whereas mean summer temperatures range from 23–26 °C^16^. Annual precipitation is between 1200–1500 mm, with a relatively higher rate of precipitation in the southern regions (including Jeju Island) compared to the central and northern regions^16^. The forests can be roughly categorized as deciduous broadleaf, temperate broadleaf, coniferous, subalpine, and alpine, with overall total species diversity of 41,483, including 5308 plant species, 1899 vertebrate species, and 22,612 invertebrate species (including 15,651 insect species)^42^.

### *M. coypus* survey and species occurrence points

A pilot survey was conducted to investigate the spatial distribution of *M. coypus* in South Korea based on earlier reports from the South Korean Ministry of Environment^43^ and previous research^10^. The survey revealed the presence of *M. coypus* across all areas of the Nakdong River basin and its drainage system, in the surrounding lakes, in the downstream area of the Namhan River, and in the Jeju Island wetlands. Detailed surveys were then performed in the Nakdong River basin and its tributaries (127° 29ʹ–129° 19ʹ E and 35° 03ʹ–37° 13ʹ N), the Namhan River and Chungjuho artificial lake located downstream (127° 50ʹ–128° 20ʹ E and 36° 50ʹ–37° 05ʹ N), and across all the Jeju Island wetlands (126° 08ʹ–126° 58ʹ E and 33° 06ʹ–34° 00ʹN) (Fig. 5). Field surveys were performed from March 2014 to December 2018.

During the surveys, all surveyors traveled on foot across river basins with an average walking speed of < 2 km·h^−1^ to monitor for footprints, living dens, feces, or make direct observations of individuals. Camera traps and *M. coypus* catching traps were installed to further confirm habitat and signs of presence. Geographic coordinates were recorded for all observations using a handheld geographical positioning system (GPS V; Garmin Inc., KS, USA). Habitat tracks were determined using the coordinate information within an area of 0.01 km^2^ in ArcGIS 10.3 (Esri, CA, USA)^7^. GPS coordinate information for some representative photographs from the *M. coypus* survey can be seen in Fig. S2. The species occurrence points for each year are shown in Fig. 1.

### Environmental variables

Nineteen bioclimatic variables^44^, in addition to distance from water, altitude, and land-cover change (Table S2), were considered important for predicting the *M. coypus* distribution in South Korea. Climatic data, including precipitation and the monthly minimum or maximum temperatures, were obtained from the Korea Meteorological Administration (KMA; https://www.kma.go.kr/eng) to estimate the current climate and future climate change scenarios of South Korea. Two greenhouse gas emission scenarios were selected for analysis, i.e., RCP 4.5 and RCP 8.5, for 2030, 2050, and 2070. The moderate and maximum emission scenarios (RCP 4.5 and RCP 8.5, respectively) correlate to predicted global mean surface temperature increases of 1.4–1.8 °C and 2.0–3.7 °C by 2100, respectively. Among the numerous available global circulation models, the HadGEM3-RA is a regional atmospheric model produced by the Met Office Hadley Centre (https://www.metoffice.gov.uk/), based on the atmospheric component of the current Earth System Model^45^. The KMA used HadGEM3-RA^45^ to create a national climate change scenario for South Korea, revealing that it tended to model small-scale features more accurately than other global circulation models, such as HADGEM2-AO, which contains more complicated topographies, lengthy and uneven coastlines, and thousands of islands along the Korean Peninsula. Accordingly, the HadGEM3-RA global circulation model was used here to determine the impacts of the RCP 4.5 and RCP 8.5 scenarios, according to the R Package *Dismo v. 1.*3^46^. Similarly to Jeon et al*.*^47^, the current climate was determined by averaging climatic data recorded between 1950 and 2000, and future climates for 2030, 2050, and 2070 were estimated from predictions for 2026‒2035, 2046‒2055, and 2066‒2075, respectively. We used a threshold of 0.7 for Pearson’s correlation coefficient^48^ for selecting bioclimatic and environmental (as described in Shin et al. ^49^ and Adhikari et al*.*^50^). Seven landcover categories (i.e., urban, cropland, forest, grassland, wetland, barren, and water) were used in the model developed by Song et al*.*^51^, based on shared socioeconomic pathways using a scenario generator in which the future population and predicted urban area were adopted for a transition matrix containing the land-cover change trends of each class. The current and future land-cover change scenarios (shared socioeconomic pathways 1) developed by Song et al.^51^ were obtained from the Korea Environment Institute MOTIVE (www.motive.kei.re.kr). Changes in the altitudinal range were quantified in the *M. coypus* distribution according to a digital elevation model. The variable distance from water included the distance from all kinds of rivers, lakes, and ponds, which were determined using the Euclidean distance option of the Spatial Analyst tool in ArcGIS Desktop 10.8. All the bioclimatic and environmental variables used in this study have a spatial resolution 0.01° (36 s), or ~ 1 km^2^, as explained by Fick and Hijmans^52^.

### Model development

SDM is a method used for predicting potential distribution of species throughout global space and time by using a correlation between the species’ geographic occurrence and its surrounding environment^53^. SDMs typically estimate the ecological niche by statistically relating environmental variables directly to species occurrence. Among the various SDM algorithms, the MaxEnt is a widely used machine learning technique for habitat suitability, which has high predictive performance using only presence data^18^. In this study, we performed MaxEnt modeling using “Biomod2” Package v.3.5.1, selecting single model MAXENT.Phillips.2^54^to predict the current and future *M. coypus* distribution in South Korea. MaxEnt exhibits a high predictive performance using presence-only occurrence data^18,55^ and is often the best option for invasive species because presence–absence survey data are usually not available, as was the case in the present study. As the MaxEnt model required background point data (e.g., pseudo-absence), we determined the background points of the study area using ArcGIS Desktop 10.8 (as suggested by Barbet-Massin et al*.*^56^). Multiple species presence points within the same ~ 1 km^2^ grid were eliminated, leaving only a single unique point per grid by adopting a spatially rarefy occurrence data tool in the ArcGIS SDM toolbox v.2.4^57^, to prevent overfitting and incorrect inflation of model performance owing to spatial autocorrelation^58^. The total species occurrence points for *M. coypus* were reduced to 2535 from 6180, and those points were used in the MaxEnt modeling. The five bioclimatic variables, distance from water, altitude, land-cover change, and the species occurrence points were used as the inputs for MaxEnt.

During modeling, the species occurrence data were randomly split into two parts, i.e., a training set and a test set, at a ratio of 3:1(as described in Adhikari et al.^59^) . The training data set was used for model fitting, which estimated the parameters of the model (model calibration). The test data set was used for model assessment, which estimated the performance of the estimated model (model validation). The other model options were run with default settings and 100 iterations, and cross-validation was maintained in the replicate runs to guarantee model accuracy^60^.

### Model evaluation and validation

The goodness-of-fit of the model was assessed, and a validation was performed with the AUC values of the receiver operating characteristic curves^61^ and the TSS^62^. The training and test data sets were used to compute the AUC values. The AUC value measures the model capacity in a model that discriminates the observed occurrences from the background data using the training and test datasets^63^. AUC is independent of dataset size (prevalence) but is limited because it weights both commission and omission errors equally, avoids the actual probability values, and is dependent on geographical extent^64^. In particular, when expanding the geographical extent beyond the present range, the AUC values increase; thus, TSS was used as an alternative criterion for model performance validation. TSS corresponds to the modeled sum of sensitivity (omission error), which is the proportion of observed presences correctly predicted, and specificity (commission error), which is the proportion of observed absences correctly predicted^61^. AUC values vary from 0‒1, whereby higher values suggest model superiority. Here, model performance was classified as poor (0.6–0.7), fair (0.7–0.8), good (0.8–0.9), or excellent (0.91–1), as suggested by Swets^65^. TSS is threshold-dependent and determines model performance by examining classification accuracy after selecting a threshold value. TSS values range between − 1 and + 1, where low values indicate an agreement that is no better than random chance, and high values represent the perfect alignment of observations and predictions^65^. In addition, a jackknife test was performed to evaluate the importance of each variable with respect to model performance^18,66^. The database and a brief conceptual flowchart of the methodology are presented in Fig. 6.

### Prediction of potential habitat and new habitat expansion and estimation of vulnerability

The MaxEnt model yielded both binary distribution maps and suitability maps of *M. coypus* under current and future climate change scenarios RCP 4.5 and RCP 8.5 for 2030, 2050, and 2070. The binary distribution maps were used to determine the suitable habitat areas across the 17 ADs of South Korea, and suitability maps were used to determine the mean and standard deviation of suitability values for each province. To calculate the distribution area, mean suitability values, and standard deviation of suitability values for each AD, the shape file representing 17 provinces of South Korea was overlain on the species distribution maps separately, and an extraction of the multiple values to points was performed using the zonal statistics of the spatial analyst tool in ArcGIS Desktop 10.8^31,67^. The vulnerability of each AD was determined and classified into three categories (low, moderate, or high) based on the mean suitability values from 0‒0.33, 0.34‒0.66, and 0.67‒1.00, respectively. We used a linear scale to classify the vulnerability and the method of Adhikari et al.^59^ with minor modifications. *M. coypus* habitat expansion was determined by differentiating between the current and future habitats using R package ‘Raster’ v3.5^68^ as described by Jeon et al.^47^.

### Ethics approval

Permission to collect species presence data are obtained from the Ministry of Environment, Republic of Korea.

## Supplementary Information

Supplementary Information 1.

Supplementary Information 2.

## Data Availability

All data generated or analyzed during this study are included in this article.

## References

[1] Kim IR (2019). Genetic diversity and population structure of nutria (*Myocastor coypus*) in South Korea. Animals.

[2] GISD*. Of the World's Worst Invasive Alien Species. Global Invasive Species Database*. http://www.iucngisd.org/gisd/100_worst.php. 100, (2021).

[3] Hong S, Do Y, Kim JY, Kim D, Joo G (2015). Distribution, spread and habitat preferences of nutria (*Myocastor coypus*) invading the lower Nakdong River, South Korea. Biol. Invas..

[4] Ojeda, R., Bidau, C. & Emmons, L. *Myocastor coypus* (*errata* version published in 2017)*. The IUCN Red List Threat. Species* (2016): e.T14085A121734257.

[5] Tsiamis K (2017). Baseline Distribution of Invasive Alien Species of Union Concern.

[6] Carter J, Leonard BP (2002). A review of the literature on the worldwide distribution, spread of, and efforts to eradicate the coypu (*Myocastor coypus*). Wildl. Soc. Bull..

[7] Kim YC (2019). Distribution and management of nutria (*Myocastor coypus*) populations in South Korea. Sustainability.

[8] Park JH (2014). The first case of *Capillaria hepatica* infection in a nutria (*Myocastor coypus*) in Korea. Korean J. Parasitol..

[9] Fratini F, Turchi BE, Ebani VV, Bertelloni F (2015). The presence of *Leptospira* in coypus (*Myocastor coypus*) and rats (*Rattus norvegicus*) living in a protected wetland in Tuscany (Italy). Vet. Arh..

[10] Lee DH, Kil JH, Kim DE (2013). The study on the distribution and inhabiting status of nutria (*Myocastor coypus*) in Korea. Korean J. Environ. Ecol..

[11] Guichón ML, Doncaster CP, Cassini MH (2003). Population structure of coypus (*Myocastor coypus*) in their region of origin and comparison with introduced populations. J. Zool..

[12] Bertolino S, Perrone A, Gola L (2005). Effectiveness of coypu control in small Italian Wetland areas. Wildl. Soc. Bull..

[13] Schertler A (2020). The potential current distribution of the coypu (*Myocastor coypus*) in Europe and climate change induced shifts in the near future. NeoBiota.

[14] Hilts DJ, Belitz MW, Gehring TM, Pangle KL, Uzarski DG (2019). Climate change and nutria range expansion in the Eastern United States. J. Wild. Manaag..

[15] Jarnevich C (2017). Evaluating simplistic methods to understand current distributions and forecast distribution changes under climate change scenarios: An example with coypu (*Myocastor coypus*). NeoBiota.

[16] Korean Metrological Administration, (2020). Korean Climate Change Assessment Report 2020.

[17] Guillera-Arroita G (2015). Is my species distribution model fit for purpose? Matching data and models to applications. Glob. Ecol. Biogeogr..

[18] Phillips SJ, Anderson RP, Schapire RE (2006). Maximum entropy modeling of species geographic distributions. Ecol. Modell..

[19] Hong S, Cowan P, Do Y, Gim JS (2016). Seasonal feeding habits of coypu (*Myocastor coypus*) in South Korea. Hystrix.

[20] Kim HS, Kong JY, Kim JH, Yeon SC, Hong IH (2018). A Case of Fascioliasis in A Wild Nutria, *Myocastor coypus* Republic of Korea. Korean J. Parasitol..

[21] Do Y, Kim JY, Im RY, Kim SB (2012). Spatial distribution and social characteristics for wetlands in Gyeongsangnam-do Province. Korean J. Limnol..

[22] IPCC. *Climate Change 2013: The Physical Science Basis. Contribution of Working Group I to the Fifth Assessment Report of the Intergovernmental Panel on Climate Change*. (2013).

[23] Sheffels, T. R. Status of Nutria (*Myocastor coypus*) Populations in the Pacific Northwest and Development of Associated Control and Management Strategies, with an Emphasis on Metropolitan Habitats, PhD Thesis (Portland State Univ., 2013).

[24] Doncaster CP, MlCOL T (1989). Annual cycle of a coypu (*Myocastor coypus*) population: Male and female strategies. J. Zool..

[25] Reggiani G, Boitani L, Stefano R (1995). Population dynamics and regulation in the coypu *Myocastor coypus* in Central Italy. Ecography.

[26] Cha Y, Cho KH, Lee H, Kang T, Kim JH (2017). The relative importance of water temperature and residence time in predicting cyanobacteria abundance in regulated rivers. Water Res..

[27] Hellmann JJ, Byers JE, Bierwagen BG, Dukes JS (2008). Five potential consequences of climate change for invasive species. Conserv. Biol..

[28] Pereira AD (2020). Modeling the geographic distribution of *Myocastor coypus* (Mammalia, Rodentia) in Brazil: Establishing priority areas for monitoring and an alert about the risk of invasion. Stud. Neotrop. Fauna Environ..

[29] Pearson RG, Dawson TP (2003). Predicting the impacts of climate change on the distribution of species: Are bioclimate envelope models useful?. Glob. Ecol. Biogeogr..

[30] Rogers CE, McCarty JP (2000). Climate change and ecosystems of the mid-atlantic region. Clim. Res..

[31] Adhikari P (2019). Potential impact of climate change on plant invasion in the Republic of Korea. J. Ecol. Environ..

[32] Welsch DJ, Smart DL, Boyer JN, Minkin P (2021). Forested Wetlands: Functions, Benefits and the Use of Best Management Practices.

[33] Borgnia M, Galante ML, Cassini MH (2000). Diet of the coypu (nutria, *Myocastor coypus*) in agro-systems of Argentinean pampas. J. Wildl. Manag..

[34] Colares IG, Oliveira RNV, Liveira RM, Colares EP (2010). Feeding habits of coypu (*Myocastor coypus* Molina 1978) in the wetlands of the Southern region of Brazil. An. Acad. Bras. Cienc..

[35] Corriale MJ, Arias SM, Bó RF, Porini G (2006). Habitat-use patterns of the coypu (*Myocastor coypus*) in an urban wetland of its original distribution. Acta Theriol..

[36] Linscombe G, Kinler N, Wright V, Chapman JA, Pursley D (1981). Nutria population density and vegetative changes in brackish marsh in coastal Louisiana. Worldwide Furbearer Conference Proceedings.

[37] Aliev F (1968). Contribution to the study of nutria migrations (*Myocastor coypus*). Saugetierkd. Mitt..

[38] Farashi A, Najafabadi MS (2017). A model to predict dispersion of the alien nutria, *Myocastor coypus* Molina, 1782 (Rodentia) Northern Iran. Acta Zool. Bulg..

[39] Vilà M (2010). How well do we understand the impacts of alien species on ecosystem services? A pan-European, cross-taxa assessment. Front. Ecol. Environ..

[40] Adhikari P (2016). Seasonal and altitudinal variation in roe deer (*Capreolus pygargus tianschanicus*) diet on Jeju Island, South Korea. J. Asia Pac. Biodivers..

[41] Koo KA, Kong WS, Nibbelink NP, Hopkinson CS, Lee JH (2015). Potential effects of climate change on the distribution of cold-tolerant evergreen broadleaved woody plants in the Korean Peninsula. PLoS ONE.

[42] National Institute of Biological Research (2014). Korean Red List of Threatened Species.

[43] Kil J (2013). Monitoring of Invasive Alien Species Designated by the Wildlife Protection Act (VII).

[44] Busby JR, Margules CR, Austin MP (1991). Bioclim, a Bioclimatic Analysis and Prediction System in Nature Conservation: Cost Effective Biological Surveys and Data Analysis.

[45] Lee I. H., Park S. H., Kang, H. S. & Cho C. H. Regional climate projections using the HadGEM3-RA in Proceedings of the 3rd International Conference on Earth System Modelling; Hamburg, Germany. 17–21 September 2012. (2012).

[46] Robert, J. H., Phillips, S., Leathwick, J. & Elith, J. *Package ‘dismo’ version 1.3.* , https://cran.rproject.org/web/packages/dismo.pdf (2021).

[47] Jeon JY, Adhikari P, Seo C (2020). Impact of climate change on potential dispersal of *Paeonia obovata* (Paeoniaceae), a critically endangered medicinal plant of South Korea. Ecol. Environ. Conserv..

[48] Dormann CF (2013). Collinearity: A review of methods to deal with it and a simulation study evaluating their performance. Ecography.

[49] Shin MS, Seo C, Lee M, Kim JY (2018). Prediction of potential species richness of plants adaptable to climate change in the Korean Peninsula. J. Environ. Impact Assess..

[50] Adhikari P (2020). Northward range expansion of southern butterflies according to climate change in South Korea. KSCCR.

[51] Song C (2018). Estimation of future land cover considering shared socioeconomic pathways using scenario generators. KSCCR.

[52] Fick SE, Hijmans RJ (2017). WorldClim 2: New 1-km spatial resolution climate surfaces for global land areas. Int. J. Climatol..

[53] Dukes JS, Mooney HA (1999). Does global change increase the success of biological invaders?. Trends Ecol. Evol..

[54] Thuiller, W., Georges, D., Gueguen, M., Engler, R. & Breiner, F. *Package ‘biomod2’: Ensemble Platform for Species Distribution Modeling*, version 3.5.1. https://cran.r-project.org/web/packages/biomod2/biomod2.pdf (2021).

[55] Elith J (2006). Novel methods improve prediction of species’ distributions from occurrence data. Ecography.

[56] Barbet-Massin M, Jiguet F, Albert CH, Thuiller W (2012). Selecting pseudo-absences for species distribution models: how, where and how many?. Methods Ecol. Evol..

[57] Brown JL (2014). SDM toolbox: A python-based GIS toolkit for landscape genetic, biogeographic and species distribution model analyses. Methods Ecol. Evol..

[58] Veloz SD (2009). Spatially autocorrelated sampling falsely inflates measures of accuracy for presence–only niche models. J. Biogeogr..

[59] Adhikari P, Lee YH, Park Y-S, Hong SH (2021). Assessment of the spatial invasion risk of intentionally introduced alien plant species (IIAPS) under environmental change in South Korea. Biology.

[60] Hong SH, Lee YH, Lee G, Lee DH, Adhikari P (2021). Predicting impacts of climate change on northward range expansion of invasive weeds in South Korea. Plants.

[61] Pearsons RG (2010). Species distribution modeling for conservation educators and practitioners. Lessons Conserv..

[62] Allouche O, Tsoar A, Kadmon R (2006). Assessing the accuracy of species distribution models: Prevalence, kappa and the true skill statistic (TSS). J. Appl. Ecol..

[63] Thuiller W, Lavorel S, Araújo MB (2005). Niche properties and geographical extent as predictors of species sensitivity to climate change. Glob. Ecol. Biogeogr..

[64] Lobo JM, Jiménez-Valverde A, Real R (2008). AUC: A misleading measure of the performance of predictive distribution models. Global. Ecol. Biogeography..

[65] Swets JA (1988). Measuring the accuracy of diagnostic systems. Science.

[66] Baldwin R (2009). Use of maximum entropy modeling in wildlife research. Entropy.

[67] Adhikari P (2018). Potential impact of climate change on the species richness of subalpine plant species in the mountain national parks of South Korea. J. Ecol. Environ..

[68] Hijmans, R. J. *et al.* Package ‘*raster’ v 3.5*: geographical data analysis and modeling. https://cran.r-project.org/web/packages/raster/raster.pdf, (2021).

