# Supplementary material for: Climate change induced habitat expansion of nutria (Myocastor coypus) in South Korea
Source: Sci Rep. 2022 Feb 28;12:3300. doi: 10.1038/s41598-022-07347-5 (PMC8885692; doi:10.1038/s41598-022-07347-5)
Supplement: Supplementary file 1 — Supplementary Information 1. [file 41598_2022_7347_MOESM1_ESM.docx]

**Supplementary Figures**

**
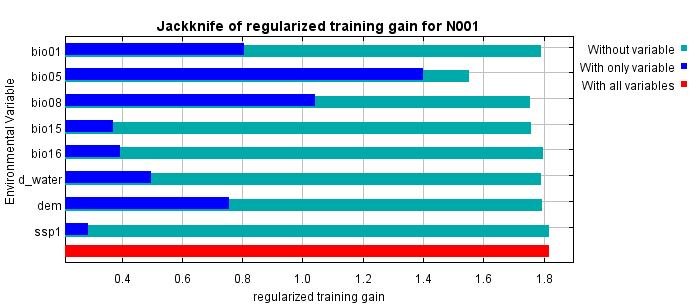
**

**Fig. S1.** Jackknife test results showing the relative contributions of variables to the current distribution. bio01, annual mean temperature; bio05, maximum temperature of warmest month; bio08, mean temperature of wettest quarter; bio15, precipitation seasonality; bio16, precipitation of wettest quarter; d_water; distance from water; dem, altitude; ssp1, land cover change.

**
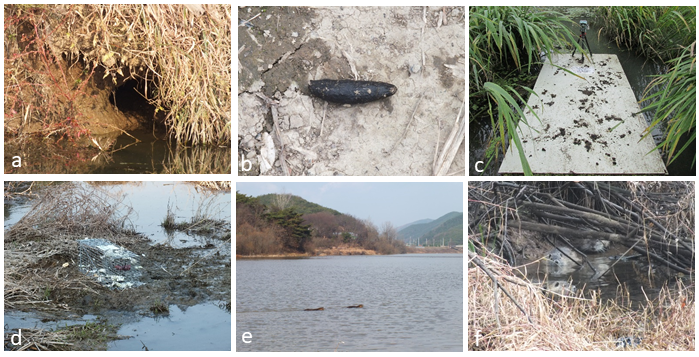
**

**Fig. S2.** Survey of nutria (*M. coypus*) in different places of South Korea. **a**, dens; **b**, feces; **c**, camera trapping; **d**, live trap setting; **e**; direct observation in the Nakdong River, Jinju; **f**, direct observation in a wetland.
