# Supplementary material for: Climate change induced habitat expansion of nutria (Myocastor coypus) in South Korea
Source: Sci Rep. 2022 Feb 28;12:3300. doi: 10.1038/s41598-022-07347-5 (PMC8885692; doi:10.1038/s41598-022-07347-5)
Supplement: Supplementary file 2 — Supplementary Information 2. [file 41598_2022_7347_MOESM2_ESM.docx]

**Table S1.** Pearson’s correlation for bioclimatic variable selection

| Variable | Bio1 | Bio2 | Bio3 | Bio4 | Bio5 | Bio6 | Bio7 | Bio8 | Bio9 | Bio10 | Bio11 | Bio12 | Bio13 | Bio14 | Bio15 | Bio16 | Bio17 | Bio18 | **d-ater** | **dem** | **SSP1** |
| --- | --- | --- | --- | --- | --- | --- | --- | --- | --- | --- | --- | --- | --- | --- | --- | --- | --- | --- | --- | --- | --- |
| Bio2 | -0.92 |  |  |  |  |  |  |  |  |  |  |  |  |  |  |  |  |  |  |  |  |
| Bio3 | -0.91 | 0.99 |  |  |  |  |  |  |  |  |  |  |  |  |  |  |  |  |  |  |  |
| Bio4 | -0.92 | 0.96 | 0.93 |  |  |  |  |  |  |  |  |  |  |  |  |  |  |  |  |  |  |
| Bio5 | -0.41 | 0.65 | 0.64 | 0.62 |  |  |  |  |  |  |  |  |  |  |  |  |  |  |  |  |  |
| Bio6 | 0.96 | -0.97 | -0.95 | -0.97 | -0.49 |  |  |  |  |  |  |  |  |  |  |  |  |  |  |  |  |
| Bio7 | -0.92 | 0.99 | 0.97 | 0.99 | 0.67 | -0.98 |  |  |  |  |  |  |  |  |  |  |  |  |  |  |  |
| Bio8 | 0.38 | -0.05 | -0.07 | 0 | 0.4 | 0.16 | -0.03 |  |  |  |  |  |  |  |  |  |  |  |  |  |  |
| Bio9 | 0.93 | -0.93 | -0.9 | -0.95 | -0.56 | 0.95 | -0.95 | 0.15 |  |  |  |  |  |  |  |  |  |  |  |  |  |
| Bio10 | 0.66 | -0.39 | -0.42 | -0.32 | 0.1 | 0.47 | -0.38 | 0.9 | 0.44 |  |  |  |  |  |  |  |  |  |  |  |  |
| Bio11 | 0.98 | -0.95 | -0.93 | -0.97 | -0.51 | 0.99 | -0.97 | 0.23 | 0.96 | 0.53 |  |  |  |  |  |  |  |  |  |  |  |
| Bio12 | 0.7 | -0.77 | -0.73 | -0.84 | -0.45 | 0.8 | -0.79 | -0.22 | 0.8 | 0.07 | 0.77 |  |  |  |  |  |  |  |  |  |  |
| Bio13 | -0.33 | 0.21 | 0.23 | 0.12 | 0.09 | -0.19 | 0.19 | -0.6 | -0.16 | -0.59 | -0.25 | 0.39 |  |  |  |  |  |  |  |  |  |
| Bio14 | 0.8 | -0.84 | -0.81 | -0.87 | -0.54 | 0.85 | -0.86 | -0.03 | 0.86 | 0.3 | 0.85 | 0.86 | 0.01 |  |  |  |  |  |  |  |  |
| Bio15 | -0.54 | 0.94 | 0.92 | 0.95 | 0.53 | -0.95 | 0.94 | -0.16 | -0.92 | -0.46 | -0.96 | -0.75 | 0.31 | -0.9 |  |  |  |  |  |  |  |
| Bio16 | 0.14 | -0.24 | -0.2 | -0.35 | -0.14 | 0.28 | -0.28 | -0.48 | 0.31 | -0.35 | 0.23 | 0.77 | 0.87 | 0.41 | -0.17 |  |  |  |  |  |  |
| Bio17 | 0.83 | -0.87 | -0.84 | -0.91 | -0.53 | 0.89 | -0.89 | -0.04 | 0.9 | 0.28 | 0.88 | 0.91 | 0.05 | 0.99 | -0.92 | 0.48 |  |  |  |  |  |
| Bio18 | -0.42 | 0.35 | 0.38 | 0.24 | 0.22 | -0.31 | 0.32 | -0.49 | -0.26 | -0.58 | -0.34 | 0.23 | 0.91 | -0.17 | 0.42 | 0.76 | -0.11 |  |  |  |  |
| Bio19 | 0.82 | -0.86 | -0.83 | -0.91 | -0.53 | 0.88 | -0.89 | -0.04 | 0.89 | 0.28 | 0.88 | 0.91 | 0.05 | 0.99 | -0.91 | 0.48 | 1 | -0.11 |  |  |  |
| **d-water** | -0.28 | 0.05 | 0.09 | -0.04 | -0.32 | -0.19 | -0.01 | -0.33 | -0.19 | -0.36 | -0.20 | 0.06 | -0.01 | 0.08 | -0.06 | 0.01 | 0.12 | 0.01 | 0.12 |  |  |
| **dem** | -0.31 | -0.08 | -0.07 | -0.04 | -0.43 | -0.21 | -0.05 | -0.41 | -0.23 | -0.41 | -0.24 | 0.21 | 0.12 | 0.15 | -0.04 | 0.17 | 0.16 | 0.14 | 0.15 | 0.16 |  |
| **SSP1** | -0.09 | 0.00 | 0.03 | -0.02 | -0.14 | -0.15 | -0.02 | -0.12 | -0.06 | -0.13 | -0.16 | 0.12 | 0.22 | -0.11 | 0.01 | 0.13 | 0.01 | 0.03 | 0.02 | 0.15 | 0.01 |

**Table S2.** List of environmental variables

| Code | Description | Unit | Source |  |
| --- | --- | --- | --- | --- |
| **Bio1** | **Annual mean temperature** | **Degrees Celsius** | **KMA** |  |
| Bio2 | Mean diurnal temperature range | Degrees Celsius | KMA |  |
| Bio3 | Isothermality (BIO2/BIO7) (* 100) | Percentage | KMA |  |
| Bio4 | Temperature seasonality | **Degrees Celsius** | KMA |  |
| **Bio5** | **Max temperature of warmest month** | **Degrees Celsius** | **KMA** |  |
| Bio6 | Min temperature of coldest month | Degrees Celsius | KMA |  |
| Bio7 | Temperature annual range | Degrees Celsius | KMA |  |
| **Bio8** | **Mean temperature of wettest quarter** | **Degrees Celsius** | **KMA** |  |
| Bio9 | Mean temperature of driest quarter | Degrees Celsius | KMA |  |
| Bio10 | Mean temperature of warmest quarter | Degrees Celsius | KMA |  |
| Bio11 | Mean temperature of coldest quarter | Degrees Celsius | KMA |  |
| Bio12 | Annual precipitation | Millimeters | KMA |  |
| Bio13 | Precipitation of wettest month | Millimeters | KMA |  |
| Bio14 | Precipitation of driest month | Millimeters | KMA |  |
| **Bio15** | **Precipitation seasonality** | **Percentage** | **KMA** |  |
| **Bio16** | **Precipitation of wettest quarter** | **Millimeters** | **KMA** |  |
| Bio17 | Precipitation of driest quarter | Millimeters | KMA |  |
| Bio18 | Precipitation of warmest quarter | Millimeters | KMA |  |
| Bio19 | Precipitation of coldest quarter | Millimeters | KMA |  |
| **d_water** | **Distance from water** | **Meter** | **This study** | |
| **dem** | **Altitude** | **Meter** | **This study** |  |
| **Ssp1** | **Land cover** | **-** | **This study** | |

KMA= Korea Meteorological Administration
